# Quality of life of alcohol dependence patients who have been having acute psychotic disorder

**DOI:** 10.1192/j.eurpsy.2021.1503

**Published:** 2021-08-13

**Authors:** V. Kuzminov

**Affiliations:** Department Of Emergency Psychiatry And Narcology, SI Institute of Neurology, Psychiatry and Narcology NAMS of Ukraine, Kharkiv, Ukraine

**Keywords:** Alcohol dependence, acute psychotic disorder, quality of life

## Abstract

**Introduction:**

Severe acute psychosis significantly alters patient’s quality of life in patients with alcohol dependence. The aim of the investigation were examination value quality of the life patients with alcohol dependence who have recently suffered of acute psychotic disorder. The factor influencing the quality of life is the psychoorganic syndrome after acute psychosis.

**Objectives:**

120 patients with alcohol dependence who had recent history of acute psychosis were examined.

**Methods:**

Psychopathological.

**Results:**

The psychorganic syndromes at these patients were investigated. The Index quality of the life in these patients was assessed due to type of the psychorganic syndromes. The dynamics of the Index quality of the life at patients with psychorganic syndrome during the treatment were described. The subjective assessment of their condition in patients with hard psychorganic syndrome was dissociated from the assessment of doctors and relatives. The explaining the characteristics of the consequences of the transferred psychotic disorder to the patients turned out to be important for overcoming anosognosia. The Index quality of the life in these patients was assessed repeatedly at the same time, there was a significant decrease in the difference in the assessment of the quality of life by patients with relatives.

**Conclusions:**

The importance of value quality of the life from the point of the patient, relatives of the patient and physician was underlined. The assesment of Index quality of the life is important important to explain the peculiarities of the postpsychotic state to the patients and their relatives in order to develop rehabilitation programs and carrying out psychotherapeutic activities.

